# A Novel Human Cytomegalovirus Locus Modulates Cell Type-Specific Outcomes of Infection

**DOI:** 10.1371/journal.ppat.1002444

**Published:** 2011-12-29

**Authors:** Mahadevaiah Umashankar, Alex Petrucelli, Louis Cicchini, Patrizia Caposio, Craig N. Kreklywich, Michael Rak, Farah Bughio, Devorah C. Goldman, Kimberly L. Hamlin, Jay A. Nelson, William H. Fleming, Daniel N. Streblow, Felicia Goodrum

**Affiliations:** 1 BIO5 Institute, The University of Arizona, Tucson, Arizona, United States of America; 2 Department of Immunobiology, The University of Arizona, Tucson, Arizona, United States of America; 3 Vaccine and Gene Therapy Institute, Oregon Health and Science University, Beaverton, Oregon, United States of America; 4 Oregon Stem Cell Center, Papé Family Pediatric Research Institute, Department of Pediatrics, and Center for Hematologic Malignancies Knight Cancer Institute, Oregon Health and Sciences University, Portland, Oregon, United States of America; Oregon Health & Science University, United States of America

## Abstract

Clinical strains of HCMV encode 20 putative ORFs within a region of the genome termed UL*b*′ that are postulated to encode functions related to persistence or immune evasion. We have previously identified UL*b*′-encoded pUL138 as necessary, but not sufficient, for HCMV latency in CD34^+^ hematopoietic progenitor cells (HPCs) infected *in vitro*. pUL138 is encoded on polycistronic transcripts that also encode 3 additional proteins, pUL133, pUL135, and pUL136, collectively comprising the *UL133-UL138* locus. This work represents the first characterization of these proteins and identifies a role for this locus in infection. Similar to pUL138, pUL133, pUL135, and pUL136 are integral membrane proteins that partially co-localized with pUL138 in the Golgi during productive infection in fibroblasts. As expected of UL*b*′ sequences, the *UL133-UL138* locus was dispensable for replication in cultured fibroblasts. In CD34^+^ HPCs, this locus suppressed viral replication in HPCs, an activity attributable to both pUL133 and pUL138. Strikingly, the *UL133-UL138* locus was required for efficient replication in endothelial cells. The association of this locus with three context-dependent phenotypes suggests an exciting role for the *UL133-UL138* locus in modulating the outcome of viral infection in different contexts of infection. Differential profiles of protein expression from the *UL133-UL138* locus correlated with the cell-type dependent phenotypes associated with this locus. We extended our *in vitro* findings to analyze viral replication and dissemination in a NOD-*scid* IL2Rγ_c_^null^-humanized mouse model. The *UL133-UL138*_NULL_ virus exhibited an increased capacity for replication and/or dissemination following stem cell mobilization relative to the wild-type virus, suggesting an important role in viral persistence and spread in the host. As pUL133, pUL135, pUL136, and pUL138 are conserved in virus strains infecting higher order primates, but not lower order mammals, the functions encoded likely represent host-specific viral adaptations.

## Author Summary

Human cytomegalovirus is a ubiquitous herpesvirus that, like all herpesviruses, establishes a life long relationship with its host through a latent infection. The molecular basis of viral latency is poorly understood, in part, because viral determinants of latency and the corresponding virus-host interactions are not well defined. We have identified a polycistronic locus encoding the pUL138 latency determinant, as well as three previously uncharacterized proteins, pUL133, pUL135, and pUL136. We have characterized this novel locus, the proteins it encodes and demonstrated the role of the locus in modulating viral replication depending on the context of infection. While this locus is dispensable for productive replication in fibroblasts, it adversely impacts virus replication in primary hematopoietic cells, suggesting a role in establishing latency. Surprisingly, the locus is required for efficient replication in primary human endothelial cells. To our knowledge this is the first demonstration of a viral locus that can have positive, negative, or null effects on viral replication depending on the context of infection. Our work defines exciting new primate strain-specific determinants mediating viral replication and latency and exemplifies the complex nature of virus-host interactions in cytomegalovirus infection.

## Introduction

Human cytomegalovirus (HCMV) is a member of the β-herpesvirus subfamily that, like all herpesviruses, persists indefinitely in infected individuals through a latent infection. HCMV persistence is associated with an increased risk of age-related pathologies including atherosclerosis [1], [2], immune senescence [3], [4], [5] and frailty [6], [7], [8] in otherwise healthy individuals. Reactivation of HCMV from latency in individuals with compromised T cell immunity, including transplant and AIDS patients, is a significant cause of morbidity and mortality [9], [10], [11], [12]. Further, HCMV is the leading cause of infectious disease-related birth defects [12], [13], [14]. The mechanisms controlling the outcome of infection, and in particular the latent infection, in the diverse cell types infected by HCMV in the human host are poorly understood. Understanding these mechanisms is essential to ultimately controlling the overt viral pathologies in individuals with weakened or insufficient T cell-mediated immunity as well as non-overt pathologies associated with viral persistence.

Clinical isolates of HCMV uniformly contain a unique region of the genome, termed UL*b*′, that encodes 20 predicted open reading frames (ORFs) [15], [16], [17]. While the actual coding potential is known for only a few UL*b*′ ORFs, these ORFs are considered dispensable for viral replication in laboratory models such as fibroblasts since laboratory-adapted strains of the virus lacking the entire UL*b*′ region replicate with increased kinetics and to increased viral yields relative to clinical strains. As such, it is postulated that UL*b*′ ORFs function in latency, immune evasion, virus dissemination in the host, or other aspects of pathogenesis. We have previously identified sequences in the UL*b*′ region of the HCMV genome encoding the *UL138* protein (pUL138) that are required for a latent infection in CD34^+^ hematopoietic progenitor cells (HPCs) infected *in vitro* [18], [19]. Disruption of the *UL138* coding sequence (cds) results in a virus that replicates with increased efficiency relative to the wild-type virus in HPCs in the absence of a reactivation stimulus. While disruption of *UL138* ablates the latent phenotype, a more robust loss of latency phenotype results from the disruption of additional UL*b*′ sequences around and including the *UL138* locus, indicating that other viral sequences in addition to *UL138* contribute to the outcome of infection in HPCs. The mechanism by which pUL138 functions in viral latency is unknown; however, it has recently been reported that the pUL138 enhances levels of tumor necrosis factor receptor (TNFR) on the cell surface [20], [21].

We have recently reported that *UL138* is part of a larger 3.6-kb polycistronic locus [22]. pUL138 is expressed from the 3′ end of three overlapping transcripts (3.6-, 2.7-, and 1.4-kb) by both canonical and stress-inducible alternative mechanisms of translation initiation [19], [22]. These transcripts encode three additional putative ORFs, *UL133*, *UL135*, and *UL136* upstream of *UL138*. We detected proteins derived from these ORFs during transient expression of *UL138* cDNAs, as well as during HCMV infection [22]. This locus may serve to coordinate the expression of pUL133, pUL135, pUL136 and pUL138 for a common function in dictating the outcome of infection in the cell.

The present study represents an initial characterization of the unique HCMV genetic locus encoding *UL133*, *UL135*, *UL136,* and *UL138*, the proteins expressed from this locus, and the role of the locus in infection. We collectively refer to this locus as the *UL133-UL138* locus. pUL133, pUL135, and pUL136 are previously uncharacterized proteins. Like pUL138, pUL133, pUL135, and pUL136 were expressed early during productive infection and ultimately localized to the Golgi apparatus. These proteins were each associated with the Golgi as integral membrane proteins with large C-terminal cytosolic domains. Despite localization to the Golgi, pUL133, pUL135, pUL136, and pUL138 were only partially co-localized. We hypothesized that the *UL133-UL138* locus functions in mediating context-dependent outcomes of infection. As would be expected for UL*b*′ sequences, the *UL133-UL138* locus was dispensable for viral replication in primary fibroblasts. We demonstrate that like *UL138*, the *UL133-UL138* locus or *UL133* alone impeded replication in CD34^+^ HPCs, consistent with a role for the encoded proteins in latency. Surprisingly, the locus augmented replication in endothelial cells. The disparate cell-type dependent phenotypes associated with the *UL133-UL138* locus correlated with differential profiles of expression from the locus in endothelial and CD34^+^ HPCs. While all four proteins were expressed in fibroblasts, we fail to detect pUL136 in endothelial cells and do not detect pUL135 or pUL136 in CD34^+^ HPCs. Further, the *UL133-UL138*_NULL_ virus exhibited an increased capacity for replication and/or dissemination in a NOD-*scid* IL2Rγ_c_^null^-humanized mouse model following stem cell mobilization relative to the wild-type virus, further suggesting an important role for the *UL133-UL138* locus in latency and reactivation. The role of individual proteins encoded by this locus in infection and latency awaits further investigation. These proteins likely represent virus adaptations to higher order primates acquired through co-speciation as the protein sequences are conserved in chimpanzee CMV (ChCMV) and to some extent in rhesus CMV (RhCMV), but are not present in CMV strains infecting lower vertebrates. Our work defines a novel locus that underscores the complexity of the virus-host interactions governing HCMV replication.

## Results

### Kinetics of *UL133-UL138* protein expression during productive infection

We have previously demonstrated the coding potential of *UL133*, *UL135* and *UL136* within the UL*b*′ region of the HCMV genome [22]. pUL133, pUL135, and pUL136 are encoded by three polycistronic transcripts of 3.6-kb, 2.7-kb and 1.4-kb, respectively, which also encode pUL138, an established determinant of HCMV latency [19], [22]. The expression of the transcripts is sensitive to inhibition of protein synthesis, but not to inhibition of viral DNA synthesis, indicating early kinetics of expression [19]. In the present study, we have characterized the expression and localization of these novel proteins as well as identified a role for this novel locus in infection.

**Figure 1:**
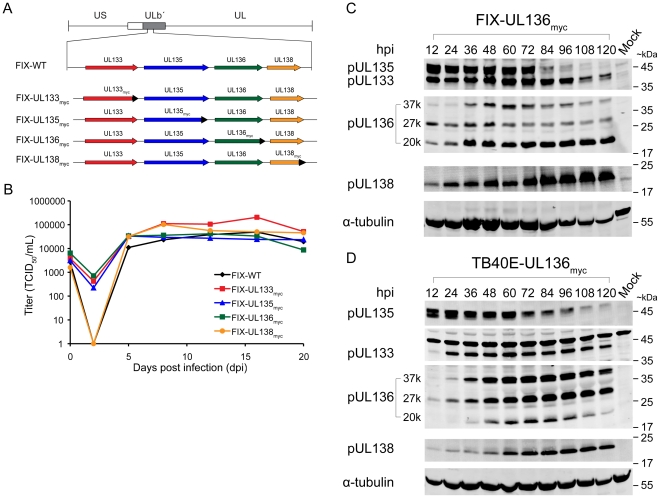
Construction and characterization of recombinant viruses expressing myc tagged ORFs. (A) Schematic of the UL*b*′ locus encoding *UL133-UL138*. The UL133_myc_, UL135_myc_, UL136_myc,_ and UL138_myc_ recombinant viruses generated in the FIX strain expressing individual ORFs with an in frame C-terminal myc epitope tag (black arrow heads) are shown. (B) Multistep growth curves for FIX recombinant viruses. Fibroblasts were infected at 0.2 MOI and virus titers measured by TCID_50_ over the time course indicated. (C–D) Kinetics of protein expression during productive infection. Fibroblasts were mock infected or infected with (C) FIX-UL136_myc_ virus or (D) TB40E-UL136_myc_ virus at an MOI of 1. Cells were harvested at 12 hr intervals and cell lysates prepared from equal numbers of cells were analyzed by immunoblotting using either rabbit anti-myc to detect pUL136 or rabbit polyclonal antibodies directed against each HCMV protein. α-tubulin serves as a loading control.

To aid in the analyses of pUL133, pUL135, pUL136, and pUL138, we constructed a series of recombinant viruses in the BAC clones of the FIX strain of CMV. We inserted the myc epitope tag in-frame at the 3′ terminus of each ORF (Figure 1A). The resulting viruses are termed FIX-UL133_myc_, FIX-UL135_myc_, FIX-UL136_myc_ [22], and FIX-UL138_myc_ [19]. The kinetics of productive viral replication in primary human embryonic fibroblasts (MRC5) infected with each of these viruses or the parental strain, FIX-WT, were measured by TCID_50_ over a time course. Despite variation in the eclipse phase, the recombinant viruses containing epitope tags replicated with kinetics and to yields reflecting that of the wild-type virus in MRC5 cells (Figure 1B and S1). The differences between the viral yields are not significant. The analogous recombinant viruses were also made in the TB40E strain of HCMV. TB40E viruses also replicated with wild-type kinetics similar to the FIX viruses.

To analyze the kinetics of pUL133, pUL135, pUL136, and pUL138 expression, MRC5 cells were infected with FIX-UL136_myc_ (Figure 1C) or TB40E-UL136_myc_ (Figure 1D) at a multiplicity of infection (MOI) of 2. FIX-UL136_myc_ was used in these experiments to aid in the detection of pUL136 because this protein is expressed at low levels in infected cells and we have not been able to generate an adequate UL136-specific antibody. Proteins were detected by immunoblotting with a monoclonal antibody specific to the myc epitope tag to evaluate pUL136 or rabbit polyclonal antibodies raised against pUL133, pUL135 or pUL138 [19], [22]. pUL133 and pUL135 have an apparent molecular mass of 39- and 43-kDa, respectively. Similar to pUL138, pUL133 and pUL135 were expressed by 12 hours post infection (hpi). pUL133 and pUL138 were expressed throughout the time course of 120 hpi, while pUL135 expression tapered off dramatically at 84 hpi. Multiple isoforms of pUL136 were detected at 37-kDa, 27-kDa, and 20-kDa, termed pUL136-37K, pUL136-27K, and pUL136-20K, respectively. An additional minor isoform was detected at 24-kDa. The 27K and 20K isoforms of pUL136 are predominantly expressed by 12 hpi and persist throughout the time course of infection. pUL136-37k exhibited slightly delayed kinetics of expression and was detected robustly at 36 hpi. Further studies are required to determine the origins of the pUL136 isoforms. Presumably, full-length pUL136 is derived from the 3.6- and 2.7-kb transcripts encoding *UL138*. We previously detected at least one smaller pUL136 isoform expressed from the 1.4-kb *UL138* transcript [22]. The relative ratios of the pUL136 isoforms differ in the TB40E infection relative to the FIX expression. This difference was consistently observed in multiple experiments; however, the reason for this difference is not known. The dynamic expression of pUL133, pUL135, and pUL136 from the *UL138* transcripts and their expression patterns are intriguing and may have important implications for their function during infection.

### pUL133, pUL135, and pUL136 localize to the Golgi apparatus

We previously determined that pUL138 localized to the Golgi apparatus during infection or transient expression [19]. To determine the subcellular localization of pUL133, pUL135, and pUL136, we infected MRC5 fibroblasts with FIX-UL133_myc_, FIX-UL135_myc_, or FIX-UL136_myc_ at an MOI of 2 and analyzed the subcellular distribution of each protein at 24 and 48 hpi by indirect immunofluorescence using a monoclonal antibody specific to the myc epitope tag. Cells were co-stained with an antibody against the Golgi marker GM130 and with DAPI to identify the nucleus. Cells infected with FIX-UL138_myc_ were used as a reference.

**Figure 2 ppat-1002444-g002:**
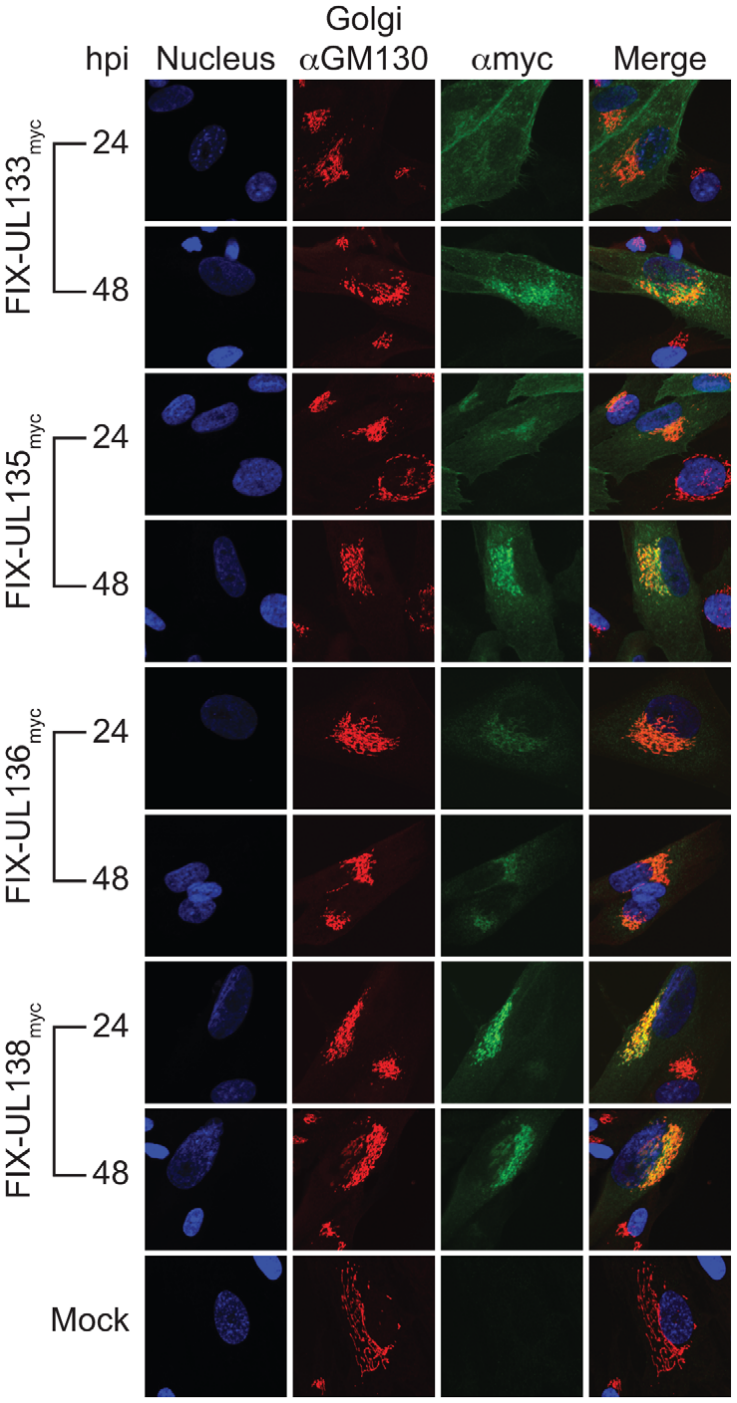
pUL133, pUL135, and pUL136 localize to the Golgi apparatus. Fibroblasts were mock-infected or infected with FIX recombinant viruses expressing myc-tagged versions of pUL133, pUL135, pUL136, and pUL138 at an MOI of 2. Viral proteins were localized by indirect immunoflurescence at 24 and 48 hpi using rabbit anti-myc antibody (71D10). A mouse monoclonal antibody specific to GM130 marks the Golgi and DAPI marks the nuclei.

By 48 hours post infection, each protein accumulated in the Golgi similarly to pUL138_myc_ (Figure 2). pUL136_myc_ resembled pUL138 in that pUL136_myc_ was Golgi associated at both 24 and 48 hpi. FIX-or TB40E-UL136_myc_ express myc-tagged versions of all pUL136 isoforms (Figure 1C and 1D); however, these forms cannot be differentiated in these experiments. pUL133_myc_ and pUL135_myc_ exhibited more diffused cellular staining at 24 hpi with predominant localization to the Golgi by 48 hpi.

### pUL133, pUL135, and pUL136 are integral membrane proteins

**Figure 3 ppat-1002444-g003:**
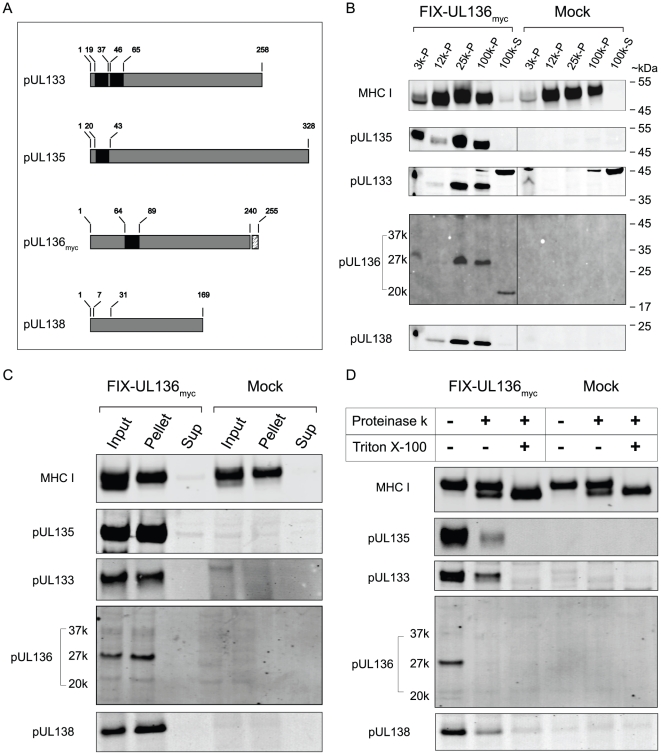
pUL133, pUL135, and pUL136 are integral membrane proteins associated with microsomal membranes. (A) Linear diagrams of indicated proteins. Amino acid positions of putative TM domains are indicated by black boxes. TM domains were predicted by multiple algorithms as part of the Mac vector software suite. The myc epitope tag on pUL136 is indicated. (B) To determine the membrane association of each viral protein, MRC5 cells were mock infected or infected with FIX-UL136_myc_ virus at an MOI of 1 were harvested at 44 hpi following 4h incubation with cycloheximide. Crude membrane fractions were prepared by differential centrifugation at the speeds indicated and analyzed for specific proteins by immunoblotting using rabbit polyclonal antibodies specific to each viral protein and the mouse monoclonal antibody specific to the myc epitope tag. (C) To determine if the viral proteins were integral membrane proteins, 25K microsomal membrane fractions were treated with 100 mM Na_2_CO_3_ for 1 h on ice and pelleted at 100,000×g for 1 h at 4^o^C. Equal amounts of input, pellet and supernatant were analyzed for specific proteins by immunoblotting as above. (D) To determine the topology of proteins in the Golgi membranes, 25K microsomal membrane fractions were treated with 0.5 µg/ml proteinase K for 45 min at 37°C in the presence or absence of 1% Triton X-100. Viral proteins were detected by immunoblotting as described above**.** MHC I was used as a control in panels B–D.

Similar to pUL138, pUL133, pUL135, and the full-length pUL136 isoform(s) are predicted to have amino terminal transmembrane (TM) domains (Figure 3A). The TM domains predicted for pUL135 and pUL136 span the membrane once, whereas pUL133 has two predicted membrane spanning domains. We investigated the membrane association of these proteins by analyzing crude membrane preparations from MRC5 cells either uninfected or infected with FIX-UL136_myc_ at an MOI of 1. Cells were treated with cycloheximide 4 hours prior to harvest to allow newly synthesized proteins to traffic to their resident compartments. 3K and 12K fractions contain cytoplasmic and nuclear membranes, respectively, whereas the 25K and 100K pellet fractions contain lighter vesicles and microsomal membranes. The 100K supernatant contains soluble proteins. Proteins were detected in each fraction using the myc antibody to detect pUL136 or polyclonal antibodies to each HCMV protein. The major histocompatability complex I (MHC I) protein was analyzed as a control using a monoclonal antibody. Similar to pUL138, pUL133 and pUL135 were concentrated in the 25K and 100K pellets indicating their association with lighter microsomal membranes (Figure 3B). This data is consistent with the localization of these proteins to the Golgi (Figure 2). pUL133, pUL135 and pUL138 also accumulated in the 3K pellet representing heavier membranes. This broad distribution may reflect trafficking of these proteins through the secretory pathway and is similar to the distribution of MHC I.

pUL136-20K was predominantly associated with the 100K supernatant, suggesting that this is a cytosolic protein. Consistent with this finding, we have previously demonstrated that smaller truncated forms of pUL136 are expressed from the 1.4-kb *UL138* transcript, which begins 300 nucleotides downstream of the full-length *UL136* start codon and lacks a predicted TM domain [22]. The pUL136-27K protein was associated with the 25- and 100-K pellets as observed for pUL133, pUL135, and pUL138, suggesting that this protein contains the N-terminal portion of the protein, including the transmembrane domain. The predicted molecular mass of the full-length pUL136 protein based on amino acid sequence is 27-kDa. Intriguingly, we did not detect pUL136-37k in experiments with a cycloheximide chase. This may be due to the low abundance and/or the narrow window of expression of this protein (Figure 1C). Alternatively, this result may be due to the rapid turnover of the protein or the sensitivity of a modification resulting in the 37-kDa mass to cycloheximide treatment.

The predicted TM domains for pUL133, pUL135, and full length pUL136 are in the N-terminus of each protein, as is the case for pUL138 (Figure 3A). In order to determine if pUL133, pUL135, and pUL136-27k are integral membrane proteins, we treated 25k microsomal membrane fractions with buffer containing 100mM sodium carbonate (Na_2_CO_3_), which is typically used to disrupt protein-protein interactions without affecting protein-lipid interactions. Following salt extraction, the pellet and the supernatant were analyzed by immunoblotting using antibodies as described for Figure 3B. pUL133, pUL135 and pUL136-27k were resistant to Na_2_CO_3_ extraction and recovered exclusively in the pellet (Figure 3C), indicating that these proteins are integral membrane proteins similar to pUL138 and MHC I [19].

To determine the orientation of pUL133, pUL135, and pUL136 in membranes, 25k microsomal membrane fractions from cells infected with FIX-UL136_myc_ were treated with proteinase K in the presence or absence of 1% Triton-X100. Lysates were analyzed by immunoblotting as described previously for Figure 3B (Figure 3D). Approximately 50% of MHC I was digested by proteinase K under native conditions to yield a lower molecular mass band as previously reported [19], [23], [24]. MHC I was completely converted to the lower molecular mass form in the presence of detergent. As previously reported, pUL138 associated with microsomal membranes was efficiently digested (60–90%) in the absence of detergent [19]. Similarly, proteinase K treatment of microsomal membranes resulted in efficient digestion of pUL133 (60–90%), pUL135 (80–90%), and pUL136 (95–100%) in the absence of detergent as observed in four independent experiments. The predicted N-terminal position of the TM domains for each of these proteins and the near complete digestion of these proteins in the absence of detergent suggest that, like pUL138, the large C-terminal domains of each protein is exposed on the cytosolic face of Golgi membranes. The less efficient digestion of pUL133 may be related to the possibility that pUL133 may span the membrane twice. Given that the majority of pUL133 is digested, we interpret these results to indicate that both the N- and C-termini of pUL133 are on the cytosolic face of 25K fraction membranes. The membrane association and the topology for each of these proteins were unchanged when expressed individually by lentivirus transduction (Figure S2). The observed topology of these proteins in Golgi membranes likely has important implications for their function during infection. Taken together, these data demonstrate that the three proteins, pUL133, pUL135, and pUL136 expressed with pUL138 from polycistronic transcripts have similar properties to pUL138 with regard to localization and membrane association.

### The *UL133-UL138* locus is dispensable for productive infection in fibroblasts

**Figure 4 ppat-1002444-g004:**
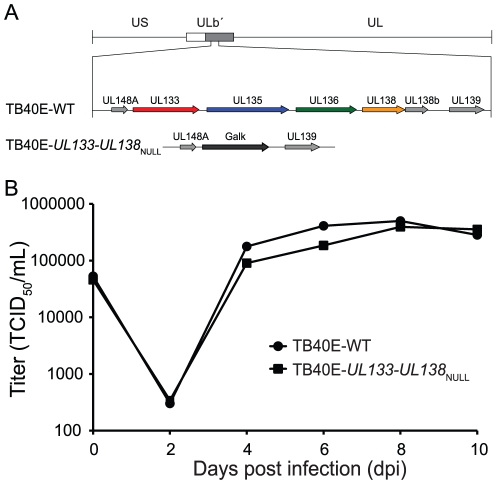
Construction and characterization of the TB40E-*UL133-UL138*_NULL_ virus. (A) Schematic of UL*b*′ locus encoding the *UL133-UL138* locus and *UL133-UL138*_NULL_ recombinant virus in which *UL133-UL138* locus replaced by Galk (black arrow) is shown. ORFs are designated by arrows. (B) To examine the replication kinetics of the TB40E-*UL133-UL138*_NULL_ virus relative to the TB40E-WT virus, MRC5 cells were infected at 0.2 MOI and virus titers measured by TCID_50_ over the time course indicated.

To address the significance of the *UL133-UL138* locus in viral infection, we constructed a recombinant virus in the TB40E strain that lacks the entire *UL133*-*UL138* locus termed TB40E-*UL133-UL138*_NULL_ (Figure 4A). The TB40E virus strain was used for these studies to analyze the role of the *UL133-UL138* locus in infection as TB40E exhibits broader tropism than the FIX strain. MRC5 cells were infected with the parental TB40E strain (TB40E-WT) or TB40E-*UL133-UL138*_NULL_ at an MOI of 0.2. Viral replication was measured over a time course by TCID_50_ (Figure 4B). As would be expected for viruses lacking UL*b*′ sequences, TB40E-*UL133-UL138*_NULL_ replicated with kinetics and to yields similar to TB40E-WT. Not unexpectedly, TB40E-*UL133-UL138*_NULL_ also replicated to similar yields as the wild-type virus at high MOI (Figure S3A). The analogous virus was also made in the FIX strain of HCMV and this virus also replicated to similar yields as the wild-type virus (Figure S3B). These results indicate that the *UL133-UL138* locus is dispensable for viral replication in fibroblasts.

### Colocalization of pUL133, pUL135, pUL136, and pUL138 during productive infection

Since each protein from the *UL133-UL138* locus localized to the Golgi (Figure 2), we next wanted to determine the extent to which the proteins co-localize in the context of productive infection. MRC5 cells were infected with TB40E-*UL133-UL138*_NULL_ at an MOI of 2 to provide the context of viral infection. At 6 hpi, cells were then co-transduced for 48 h with four lentivirus contructs expressing pUL133, pUL135, pUL136, and pUL138, each with a different carboxy terminal epitope tag. Antibodies specific to the Flag (FLAG), hemagglutinin (HA), myc, or glu-glu (EE) epitope tags were conjugated with Quantum dots and used to label pUL133_FLAG_, pUL135_HA_, pUL136_myc_, and pUL138_EE_, respectively. When expressed independently, pUL133_FLAG_, pUL135_HA_, pUL136_myc_, and pUL138_EE_ localized in part to a perinucler region resembling the Golgi, implying that the localization of these proteins to the Golgi did not require other viral proteins or the context of infection (Figure S4). Similar to infection (Figure 2), both pUL133_FLAG_ and pUL135_HA_ exhibited a more diffuse localization showing both cell surface and perinuclear staining resembling the Golgi (Figure S4). Importantly, Figure S4 demonstrates that there was no appreciable background or bleed through of these signals in each of the five channels.

**Figure 5 ppat-1002444-g005:**
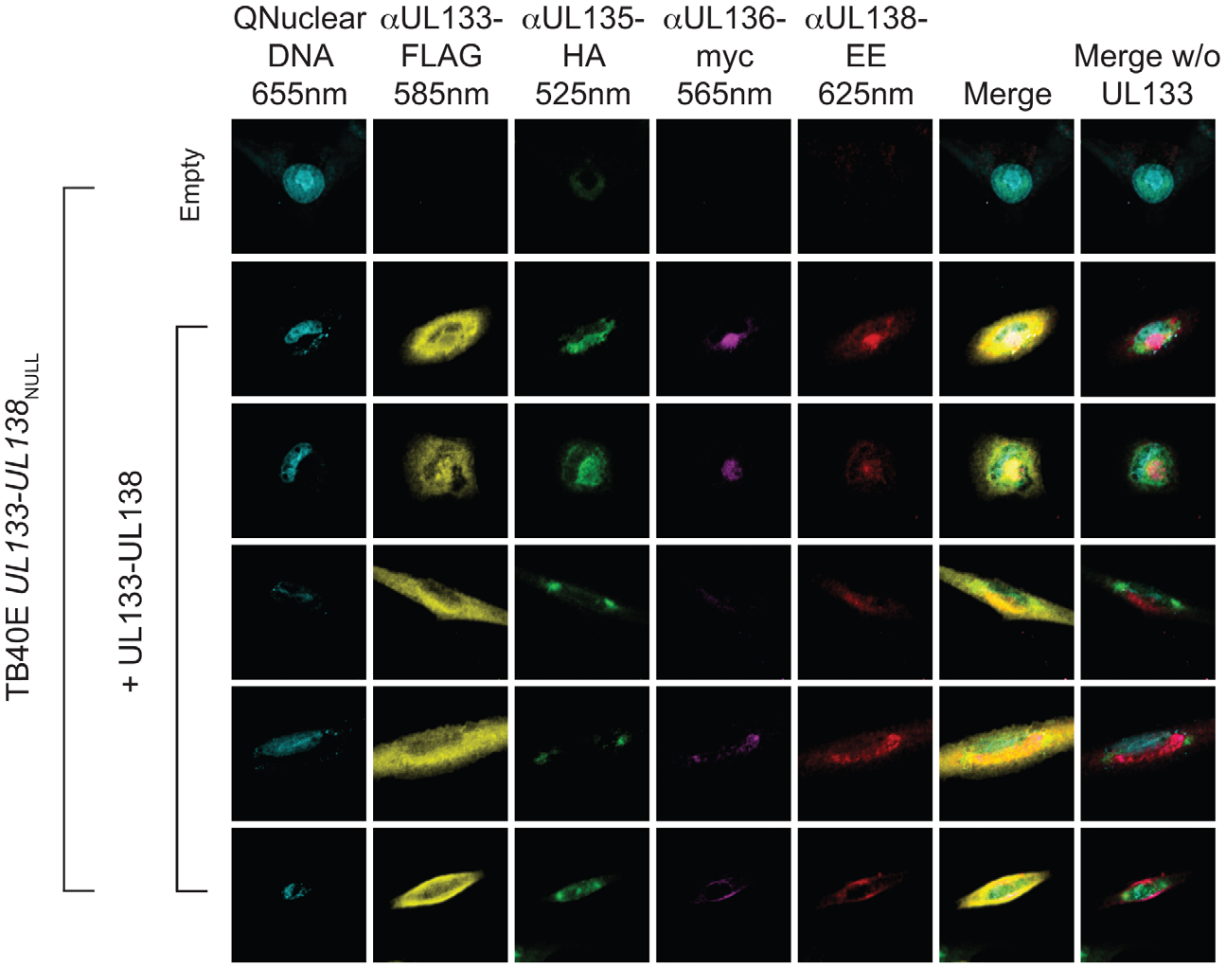
Colocalization of pUL133, pUL135, pUL136 and pUL138 in the Golgi apparatus. MRC5 cells were infected with TB40E-*UL133-UL138*_NULL_ and co-transduced with lentiviruses encoding pUL133_FLAG_, pUL135_HA_, pUL136_myc_, and pUL138_EE_ or an empty lentivirus at an MOI of 2. Proteins were co-localized by direct immunofluorescence 48 hpi using monoclonal antibodies specific to each epitope tag that had been directly conjugated to Quantum dots of 525nm (HA); 565nm (Myc); 585nm (FLAG); and 625nm (EE). Cell nuclei are indicated by Qnuclear staining. Localization was visualized using a Ziess 510 Meta Confocal microscope. Images for five representative cells are shown in the five rows below empty.

When expressed together, there was substantial overlap in the signals for all four proteins (Figure 5). Due to the high level of pUL133_FLAG_ expression, merged images are shown of all viral proteins with and without pUL133_FLAG_. pUL136_myc_ and pUL138_EE_ exhibited the greatest overlap in their localization pattern. There were some notable differences in staining where pUL135_HA_ appears to be excluded from perinuclear regions strongly stained with pUL133_FLAG_ and pUL138_EE_. It is interesting that regions of pUL135_HA_ staining are typically juxtaposed to regions of intense pUL133_FLAG_ and pUL138_EE_ staining. Importantly, no signal was detected in infected cells transduced with an empty vector, demonstrating the specificity of the antibodies and that these antibodies were not bound by viral Fc receptors. The significance of these patterns and regions of overlapping and non-overlapping protein localization awaits further investigation.

### Expression of the *UL133-UL138* locus in CD34^+^ HPCs

**Figure 6:**
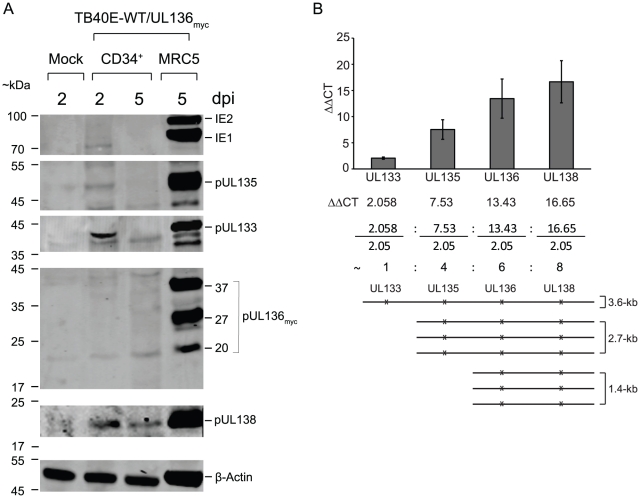
*UL133-UL138* locus expression in CD34^+^ HPCs. CD34^+^ HPCs infected with TB40E-WT or TB40E-UL136_myc_ at MOI 2.0. Pure populations of infected CD34^+^ cells were maintained in LTBMC. (A) Lysates prepared from equal cell numbers at 2 and 5 days post infection (dpi) were analyzed by immunoblotting using either mouse anti-myc antibody to detect pUL136 or rabbit polyclonal antibodies directed against pUL133, pUL135, or pUL138. β-actin was used as a loading control. (B) RNA isolated from infected CD34^+^ HPCs was analyzed at 2 dpi by qRT-PCR. The relative target levels for *UL133*, *UL135*, *UL136*, and *UL138* transcripts were determined using the ΔΔCT equation and are expressed here as a fold over β-actin that was used as an internal reference. The ratio of 3.6-, 2.7-, and 1.4-kb transcripts is indicated. A schematic representative of the relative abundance of each transcript is shown. The bars represent an average of three replicates where the standard deviation is shown.

Given the role of pUL138 in latency, the other proteins expressed from the *UL133-UL138* locus may function to cooperate with pUL138 in establishing a latent infection in CD34^+^ cells. We first analyzed protein expression from the *UL133-UL138* locus in infected CD34^+^ HPCs. CD34^+^ HPCs, freshly isolated from umbilical cord blood were infected with TB40E-WT or TB40E-UL136_myc_ at an MOI of 2. Pure populations of infected (GFP positive) CD34^+^ cells were isolated by fluorescent activated cell sorting (FACS) and seeded into long-term bone marrow cultures (LTBMC) over a stromal support. Whole cell lysates were prepared at 2 and 5 days post infection (dpi) and analyzed for protein expression by immunoblotting using polyclonal antisera to HCMV proteins, the monoclonal antibody specific to IE1 and IE2, or a monoclonal antibody recognizing the myc epitope tag. Representative blots are shown from five independent experiments. We detected transient expression of IE1, which did not persist past 2 dpi (Figure 6A). IE1 transcript expression in HPCs has been detected previously [19], [25], [26]. Similarly, pUL133 was detected at 2 dpi but not at 5 dpi. As previously observed, pUL138 was expressed in CD34^+^ HPCs at 2 and 5 dpi [19]. By contrast, we detected very low to undetectable levels pUL135 or pUL136 in these cells under conditions where the expression of pUL138 was readily detected. While we cannot exclude the possibility that pUL135 and pUL136 are expressed in CD34^+^ HPCs, their relative abundance is substantially diminished compared to infected fibroblasts and we consistently fail to detect these proteins in CD34^+^ HPCs with our current tools. These results indicate that expression of individual proteins from the *UL133-UL138* locus may be differentially modulated based on the context of infection. Given the low levels of expression of pUL135 and pUL136 detected in CD34^+^ HPCs, it is uncertain as to what role these proteins may play in the establishment and maintenance of latency.

The differential expression of *UL133-UL138* locus proteins in CD34^+^ cells may be due to the relative abundance of transcripts encoding pUL138. As we have shown previously, pUL138 can be expressed from 3.6-, 2.7-, and 1.4-kb transcripts, although it appears to be most efficiently expressed from the 1.4-kb transcript [22]. By contrast, pUL133 and pUL135 are expressed only from the 3.6- and 2.7-kb transcripts, respectively. We reasoned that if the 1.4-kb transcript was expressed more abundantly in CD34^+^ HPCs than the 3.6- and 2.7-kb transcripts, this could explain the enhanced levels of pUL138 expression. RNA was isolated from infected CD34^+^ HPCs at 2 dpi and the 3.6-, 2.7-, and 1.4-kb transcripts were quantitated by real time reverse-transcriptase PCR (qRT-PCR) using primers specific to *UL133*, *UL135*, *UL136* or *UL138*. As a control, we used TB40E-*UL133-UL138*_NULL_ where none of these transcripts are expressed. We detected significant levels of 3.6-, 2.7- and 1.4-kb transcripts compared to control infection. Transcript levels of IE1 and IE2 were nearly identical in cells infected with both TB40E-WT and TB40E-*UL133-UL138*_NULL_ (fold change of 1.3 and 1.4, respectively; data not shown). The 3.6-, 2.7-, and 1.4-kb transcripts were present in 1∶3∶3 ratio, respectively (Figure 6B). While these results could explain the higher level of pUL138 expression, they do not explain the absence of pUL135 and pUL136 since the ratio of 2.7- to 1.4-kb transcripts is 1. Therefore, translational regulation of these transcripts may also play a role in protein abundance.

### The *UL133-UL138* locus suppresses viral replication in CD34^+^ HPCs

**Figure 7:**
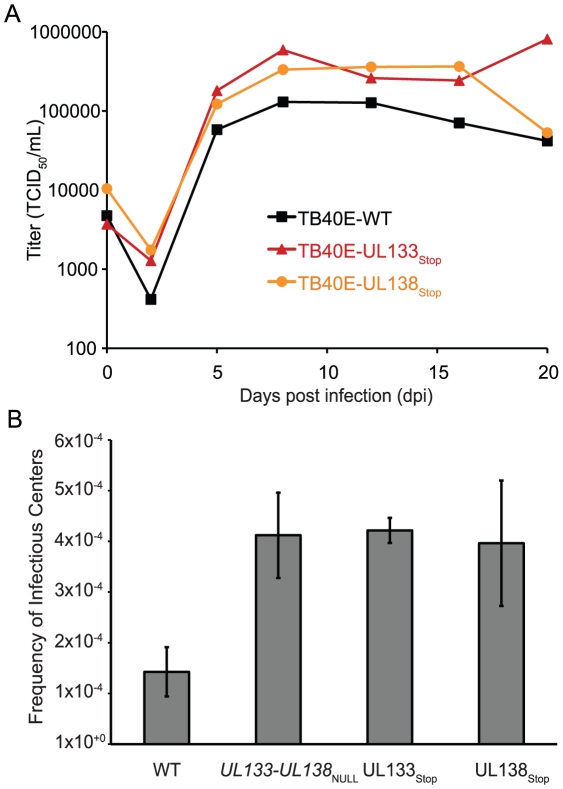
*UL133-UL138* locus function in CD34^+^ HPCs. (A) Multistep growth curves for TB40E recombinant viruses. Fibroblasts were infected at 0.2 MOI and virus titers measured by TCID_50_ over the time course indicated. (B) CD34^+^ HPCs infected with TB40E-WT, TB40E-*UL133-UL138*_NULL_, TB40E-UL133_Stop_ or TB40E-UL138_Stop_ viruses at MOI 2.0. Pure populations of infected CD34^+^ cells were maintained in LTBMC for 10 dpi after which lysates from infected CD34^+^ HPCs were serially diluted onto MRC5 cells plated in 96 well dishes. Infectious centers formation was determined from the number of GFP^+^ wells at each dilution 15–20 days later. The frequency of infectious centers was calculated using ELDA software. The data shown is an average of three independent experiments. TB40E-*UL133-UL138*_NULL_, TB40E-UL133_Stop_ and TB40E-UL138_Stop_ viruses replicated with 5-fold greater efficiency compared to TB40E-WT (p = 0.0043, p = 0.0005 and 0.0148, respectively).

We next sought to determine the role of the *UL133-UL138* locus in latency. We have previously demonstrated a role of pUL138 in the FIX strain for promoting the latent infection [18], [19]. For a relative comparison, we generated a virus using the TB40E strain, TB40E-UL138_Stop,_ where *UL138* was disrupted by the substitution of the initiator codon with a stop codon. Further, as pUL133 expression was reliably detected in infected CD34^+^ HPCs (Figure 6A), we generated a virus containing a similar disruption in *UL133*. We analyzed the replication of these recombinants relative to TB40E-WT in MRC5 cells using multi-step growth curves (Figure 7A). Similar to FIX strains containing disruptions in *UL138*, TB40E-UL133_Stop_ and TB40E-UL138_Stop_ replicated with slightly enhanced yields relative to TB40E-WT [19]. These results demonstrate that *UL133*, like *UL138,* is dispensable for viral replication in fibroblasts.

To analyze latency, CD34^+^ HPCs were infected with TB40E-WT, TB40E-*UL133-UL138*_NULL_, TB40E-UL133_Stop_, or TB40E-UL138_Stop_ at an MOI of 2. Pure populations of infected CD34^+^ cells were incubated in LTBMC for 10 dpi. Cell lysates were analyzed by an infectious centers assay to determine the number of cells required to form an infectious center [18], [26]. This assay is a measure of virus replication, but distinct from a plaque forming or TCID_50_ assays. The infectious centers assay is appropriate for these measures because each infected CD34^+^ HPC does not go on to produce virus upon reactivation. It is thought that the differences between viruses with regards to the establishment of or reactivation from latency is the number of infected cells producing virus as opposed to the yield of virus per cell [18], [26]. TB40E-*UL133-UL138*_NULL_ (p = 0.0043), TB40E-UL133_Stop_ (p = 0.0005), and TB40E-UL138_Stop_ (p = 0.0143) replicated with increased efficiency in HPCs relative to the wild type virus (Figure 7B), producing 5-fold greater infectious centers compared to cells infected with the wild-type virus. Similar to previous findings for *UL138*, these data suggest a role for *UL133, UL138,* and the entire *UL133-UL138* locus in suppressing viral replication, presumably for the latent infection in HPCs [18], [19]. Further work is required to determine the individual contributions of these and other *UL133-UL138* locus proteins to viral replication or latency in this model.

### The *UL133-UL138* locus is required for efficient viral replication in primary endothelial cells

**Figure 8:**
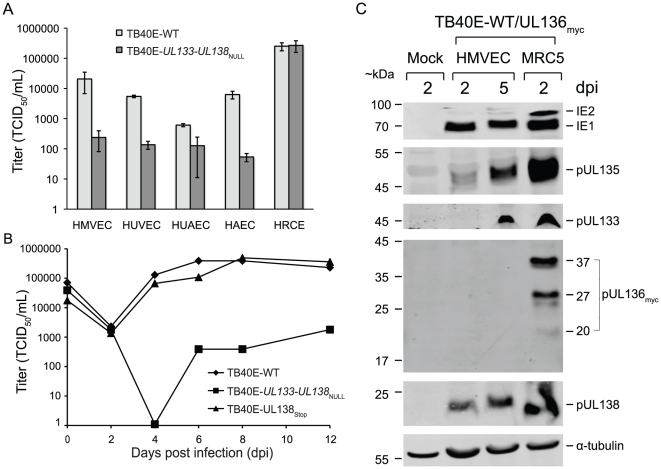
Expression and function of *UL133-UL138* locus in primary endothelial cells. (A) Four endothelial cell types (HMVEC, HUVEC, HUAEC or HAEC) and one epithelial cell type (HRCE) were infected with TB40E-WT or TB40E-*UL133-UL138*_NULL_ at MOI 0.1. At 10 dpi, virus titers in lysates were determined by TCID_50_ on MRC5 cells. (B) HMVEC-L cells infected with TB40E-WT, TB40E-*UL133-UL138*_NULL_ or TB40E-UL138_Stop_ at MOI 0.1 were harvested at indicated times post infection to analyze multi-step replication. Virus titers were determined by TCID_50_ on MRC5 cells. (C) HMVEC cells infected with TB40E-WT or TB40E-UL136_myc_ at MOI 2.0 were harvested at 2 and 5 days post infection (dpi) and lysates prepared from equal numbers of cells were analyzed by immunoblotting using either mouse anti-myc antibody to detect pUL136 or rabbit polyclonal antibodies directed against each *UL133-UL138* locus protein.

HCMV infects a wide array of cell types in the human host. UL*b*′ genes are predicted to encode functions that mediate viral replication, dissemination, and persistence in the host. To determine if the *UL133-UL138* locus is important for viral replication in other cell types, we compared viral yields in endothelial and epithelial cells infected with TB40E-WT or TB40E-*UL133-UL138*_NULL_ (Figure 8A). We chose 3 different primary human endothelial cell types (microvascular lung, HMVEC; umbilical vein, HUVEC; aortic, HUAEC), one endothelial cell line (HAEC), and one primary human renal epithelial cell type HRCE. In each case, cells were infected with an MOI of 0.1 and cell lystates were harvested 10 dpi. Surprisingly, TB40E-*UL133-UL138*_NULL_ exhibited a modest to severe replication defect (5–200 fold) in all endothelial cell types analyzed. By comparison, TB40E-*UL133-UL138*_NULL_ replicated similarly to TB40E-WT in the epithelial cell type tested.

To further explore the role of the *UL133-UL138* locus in endothelial cells, we analyzed multi-step replication of UL133-UL138_NULL_ in HMVECs. HMVECs were infected with TB40E-WT, TB40E-*UL133-UL138*_NULL_ or TB40E-UL138_Stop_ at an MOI of 0.2 and whole cell lysates analyzed for virus production over a time course following infection by TCID_50_ (Figure 8B). TB40E-*UL133-UL138*_NULL_ exhibited a 2-log defect in replication relative to TB40E-WT. This defect in replication was not due to a failure of the mutant virus to enter or spread in HMVEC cells based on the initial number of infected (GFP^+^) cells and the formation of plaques, respectively (data not shown). Further, this defect cannot be overcome by infecting cells at higher multiplicities (MOI of 2; data not shown). Viruses lacking only *UL138* exhibited no defect relative to TB40E-WT, suggesting pUL138 is not required for replication in these cells.

We next analyzed protein expression from the *UL133-UL138* locus in endothelial cells. HMVECs were infected at an MOI of 2 and whole cell lysates were analyzed for pUL133, pUL135, pUL136 and pUL138 expression at 2 and 5 dpi by immunoblotting. As a control for infection, we analyzed the expression of the IE1 and IE2 proteins. We detect IE1 in HMVECs, but IE2 is consistently expressed at low to undetectable levels in these cells (Figure 8C). With regards to the *UL133-UL138* locus, we readily detected expression of pUL135 and pUL138 at 2 dpi. pUL133 was detected, but only at the 5 dpi time point. Expression of pUL136 was undetectable in each of three independent infections. The failure to detect pUL136 may be due to the variability in expression of the pUL136 isoforms or in the inherent instability of pUL136 (Cicchini and Goodrum, unpublished results).

### The *UL133-UL138* locus modulates virus reactivation and dissemination *in vivo*

Given the three cell type-dependent replication phenotypes associated with the *UL133-UL138* locus, we analyzed viral replication and dissemination in a NOD-*scid* IL2Rγ_c_^null^-humanized mouse model. This model represents the only animal model to effectively study HCMV infection parameters including of latency and reactivation [27]. NOD-*scid* IL2Rγ_c_^null^ mice were engrafted with human CD34^+^ HPCs. The huCD34^+^-engrafted mice were transfused with human fibroblasts infected with TB40E-WT or TB40E-*UL133-UL138*_NULL_ or uninfected fibroblasts as a negative control (8 mice per experimental group). At 4 weeks post infection, four mice in each group were treated with granulocyte-colony stimulating factor (G-CSF) and AMD-3100 to induce stem cell mobilization and viral reactivation. At two weeks post mobilization, we measured viral genome loads in bone marrow and spleen tissues by quantitative TaqMan PCR with probes and primers specific for HCMV US28.

**Figure 9:**
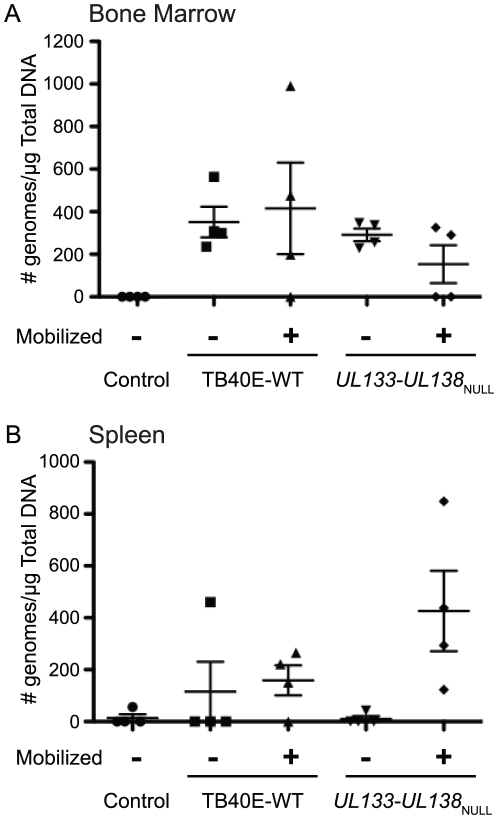
Analysis of *UL133-UL138*_NULL_ infection in humanized mice. NOD-*scid* IL2Rγ_c_^null^ mice were engrafted with human CD34^+^ HPCs and then transfused with human fibroblasts infected with TB40E-WT or TB40E-*UL133-UL138*_NULL_ virus. Uninfected mice served as negative controls (n = 4). At 4 weeks post infection, mice were treated with G-CSF and AMD-3100 or left untreated (n = 4/group) for two weeks and then euthanized for tissue harvesting and analysis. Total genomic DNA was isolated from (A) bone marrow or (B) or spleen and analyzed for the presence of viral DNA by quantitative PCR using primers and probe specific for HCMV-US28. Data is represented as viral DNA copy number/µg of total DNA. Viral DNA loads in the bone marrow for WT and *UL133-UL138*_NULL_ infections were nearly equal (p = 0.17 and 0.18 for unmobilized and mobilized mice, respectively). Viral loads in the spleen were greater in the mobilized *UL133-UL138*_NULL_-infected mice compared to mobilized WT-infected mice (p = 0.08) and compared to the unmobilized *UL133-UL138*_NULL_-infected mice (p = 0.001).

HCMV genomic DNA was detected in the bone marrow of both wild type and *UL133-UL138*_NULL_ infected non-mobilized mice (351 copies/µg DNA for TB40E-WT vs. 291 copies/µg for TB40E-*UL133-UL138*_NULL_; p = 0.17; not significant) and did not increase significantly upon mobilization (Figure 9A). Both viruses showed an increase in splenic viral DNA loads following mobilization suggesting that cells infected with both viruses were disseminated to the spleen (Figure 9B). However, mobilization of the mice infected with TB40E-*UL133-UL138*_NULL_ resulted in a 2- to 3-fold higher levels of viral DNA load in the spleen compared to wild-type-infected animals (p = 0.08). Mice infected with TB40E-WT had an overall 1.4-fold increase in spleen viral DNA load following mobilization versus a 43-fold increase in TB40E-*UL133-UL138*_NULL_ infected mice. Low levels of viral DNA were detected in the spleens of unmobilized mice infected with either TB40E-WT or *UL133-UL138*_NULL_ because infected cells do not efficiently traffic out of the bone marrow in the absence of mobilization. These data indicate that the *UL133-UL138* locus is important for modulating viral replication, reactivation or dissemination in this model.

### The *UL133-UL138* locus encodes proteins unique to human and chimpanzee CMV

In an effort to understand the possible function of the proteins encoded from the *UL133-UL138* locus, we searched the known protein sequence databases for protein sequence similarity using BLASTpsi (http://blast.ncbi.nlm.nih.gov/Blast.cgi). Further, we used PHOG (http://phylofacts.berkeley.edu/orthologs/) to predict super-orthologs based on phylogenetic analysis [28]. Finally, we used Phyre (http://www.sbg.bio.ic.ac.uk/~phyre/) to predict three dimensional structure using homology modeling, which does not rely on conservation of protein sequence [29]. No cellular or viral homologs were identified by any of these bioinformatics methods for any of the *UL133-UL138* locus cds with the exception of HHV-5/CMV orthologues (data not shown). Further, no protein structures could be predicted.

Due to the lack of identifiable protein structure, we next analyzed these proteins for regions of disorder using Disopred2 (http://bioinf.cs.ucl.ac.uk/disopred). This algorithm predicted large regions of disorder across pUL133, pUL135, pUL136 and pUL138 suggesting that these are intrinsically disordered proteins (data not shown). Intrinsically disordered proteins typically adapt structure through their interactions and often interact with a large number of proteins [30]. These analyses indicate that the *UL133-UL138* locus proteins are unique to CMV and, as such, will require further molecular and biochemical studies to understand their role in infection.

**Figure 10:**
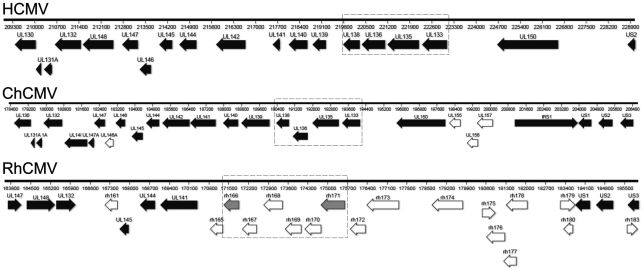
Orthologues to *UL133-UL138* locus proteins. The UL*b*′ region from HCMV, ChCMV, and RhCMV were aligned to identify orthologues of pUL133, pUL135, pUL136, and pUL138. Conserved orthologues of ChCMV and RhCMV are shown with black arrows.

To determine the extent of conservation of the *UL133-UL138* locus within CMV orthologues, we aligned the UL*b*′ sequences available from NCBI for HCMV (strain TB40E; Accession: EF99921.1; GI: 157779983), ChCMV (strain heberling; Accession: NC_003521.1; GI: 20026600) and RhCMV (strain 68-1; Accession: NC_006150.1; GI: 51556461) (Figure 10). Of note, sequences with similarity to the UL*b*′ region were identified only in ChCMV and RhCMV and not in any CMVs of lower mammals for which a sequence is known. Orthologues to each gene encoded within the *UL133-UL138* locus are present in ChCMV. The ChCMV orthologues for the strains aligned share 44.2%, 46.7%, 53.8% and 56.7% similarity at the amino acid level with pUL133, pUL135, pUL136, and pUL138, respectively. In RhCMV, the UL*b*′ region is positionally conserved. However, few RhCMV genes in this region share substantial sequence identity with HCMV UL*b*′ genes [31]. RhCMV Rh166 ORF shows similarity to both HCMV pUL133 (26.6%) and pUL138 (35%). In addition, the RhCMV Rh171 ORF also shows similarity to HCMV pUL133 (27.6%). Hence, Rh166 and Rh171 might represent the orthologues for HCMV pUL138 and pUL133, respectively, though the exact corresponding homologues are not clear from the analyses performed. No significant identity between UL135 and UL136 of HCMV and RhCMV proteins was observed.

## Discussion

We have identified and characterized a novel locus within the UL*b*′ region of the HCMV genome that encodes three proteins, pUL133, pUL135, and pUL136, in addition to the pUL138 latency determinant [22]. The pUL133, pUL135, and pUL136 proteins have not been previously characterized. Proteins encoded by the *UL133-UL138* locus predominantly localize to Golgi membranes (Figure 2, 3 and 5) with large C-terminal domains exposed on the cytosolic face of the membranes (Figure 3). *UL133-UL138* locus proteins exhibit overlapping localization, but did not completely co-localize (Figure 5). The profile of expression of the individual proteins from the *UL133-UL138* locus varied substantially depending on the context of infection (Figure 1C–D, 6A, 8C). As would be expected for UL*b*′ sequences, the *UL133-UL138* locus was dispensable for viral replication in fibroblasts (Figure 4 and S3). Disruption of the *UL133-UL138* locus resulted in a virus, *UL133-UL138*_NULL,_ with increased frequency of infectious centers formation in CD34^+^ HPCs relative to the wild-type virus (Figure 7B), consistent with a failure to establish a latent infection. Intriguingly, *UL133-UL138*_NULL_ exhibited a severe replication defect in primary human endothelial cells (Figure 8A and 8B). These three distinct context-dependent phenotypes indicate that the *UL133-UL138* locus may mediate context-dependent outcomes of infection. The mechanism by which the *UL133-UL138* locus contributes to cell-type dependent outcomes of infection awaits further investigation. Importantly, *UL133-UL138*_NULL_ exhibited increase replication and dissemination in a NOD-*scid* IL2Rγ_c_^null^-humanized mouse model (Figure 9), indicating a role for this locus in mediating viral replication, latency or dissemination *in vivo*. Further, as the *UL133-UL138* locus is unique to CMV strains of higher order primates, we predict that these proteins represent viral adaptations to infection and persistence in the primate host (Figure 10).

The UL*b*′ region of the HCMV genome was first recognized over a decade ago [15]. As this region is unique to CMV strains infecting primates and is dispensable for viral replication in fibroblasts, the most common model for productive viral replication, it presents significant challenge for research and has been understudied. The genes in this region are postulated to encode viral adaptations to the host, involved in immune evasion, pathogenesis, or viral persistence or latency. The coding potential of genes in the UL*b*′ region has been shown for only a few of the 20 putative ORFs. These include *UL138*, *UL141*, *UL142*, *UL144*, and *UL146*. *UL138* encodes a protein that is required, but not sufficient, for the latent infection in HPCs infected *in vitro* [18], [19]. It has been recently demonstrated that pUL138 functions to regulate cell surface levels of TNFR [20], [21]. *UL141* [32] and *UL142* [33] encode proteins to evade elimination by natural killer cells. *UL142* is expressed late in infection and encodes a MHC class I related molecule, which renders the cells resistant to NK mediated cell lysis. *UL144* functions as a tumor necrosis factor (TNF) homolog that activates NFkB, which in turn enhances the expression of CCL22, a chemokine, which attracts Th2 and T regulatory cells. Thus *UL144* may help the virus evade immune surveillance by enhancing the Th2 response while subverting the Th1 response [34], [35]. *UL146* encodes a viral CXCL chemokine that binds the IL-8 receptor to enhance neutrophil chemotaxis and degranulation [36]. Another putative CXC homologue, *UL147*, has been identified [36]; however, the coding potential of this ORF and its function has not been explored. Most recently, we have extended our studies on pUL138 to characterize the protein coding capacity of the *UL133*, *UL135*, and *UL136* ORFs, encoded on polycistronic transcripts with *UL138* [22]. The individual functions of pUL133, pUL135, and pUL136 have not yet been determined. The sequence analyses of these proteins do not indicate any obvious sequence motifs or functional domains that suggest protein function. The fact that these proteins share similar cellular and biochemical properties (Figure 2, 3 and 5) to that of pUL138 suggests that they may function together during infection. We hypothesize that these proteins may function with pUL138 in modulating the outcome of infection in a context dependent manner. Our ongoing research focuses on determining the functions of each of these proteins during HCMV infection.

The proteins derived from the *UL133-UL138* locus share identity only with other HHV-5 orthologues. While the proteins encoded by the *UL133-UL138* locus are conserved in chimpanzee CMVs, orthologues have not been identified in cytomegalovirus strains infecting lower mammals, including RhCMV and MCMV [31]. In RhCMV, the UL*b*′ region is positionally conserved, but few proteins have considerable sequence identity (Figure 10) [31]. Both *UL133* and *UL138* share moderate similarity to *rh166* while *UL133* also shows weak homology to *rh171*. We propose, given the position of the Rh166 and Rh171 ORFs and the moderate conservation of sequence, that these ORFs represent orthologues of pUL133 and pUL138 of HCMV. No orthologues were identified for pUL135 or pUL136, as previously reported [31]. These observations suggest that *UL133-UL138* locus resulted from co-speciation in higher order primates, and suggest an intriguing possibility that these proteins engage in virus-host interactions that are highly adapted to the host species.

Our studies indicate, as would be expected for UL*b*′ sequences, that the *UL133-UL138* locus was dispensable for replication in cultured fibroblasts (Figure 4). However, the locus augmented replication in primary endothelial cells (Figure 8) and impeded replication in CD34^+^ HPCs (Figure 7B). Further, profiles of gene expression from the UL133-UL138 locus varied depending on the cell type infected (compare Figure 1C and D to [Fig ppat-1002444-g002] [Fig ppat-1002444-g003] [Fig ppat-1002444-g004] [Fig ppat-1002444-g005] 6A and 8C). For example, pUL135 was not detected in CD34^+^ HPCs, but was expressed efficiently in endothelial cells and fibroblasts, while pUL136 could not be detected in endothelial cells of CD34^+^ HPCs. This finding suggests that the ultimate outcome of infection may rely on the profile of protein expression from the *UL133-UL138* locus in individual contexts of infection. As pUL135 and pUL136 are not expressed in HPCs, they may not be required for establishing or maintaining latency, but may function in some other aspect such as reactivation.

Interactions between proteins encoded by the *UL133-UL138* locus or with unique cell-type dependent host factors may underlie the role of this locus in mediating cell-type specific infection outcomes. Preliminary studies aimed at understanding the function of *UL133-UL138* locus proteins have revealed a complex network of interactions and positive and negative acting proteins (Umashankar, Petrucelli, Rak, and Goodrum, unpublished results). The localization of *UL133-UL138* locus proteins to the Golgi (Figure 2 and 5) and their orientation in the membranes (Figure 3) is certainly foretelling of the function of these proteins. Proteins localized in the Golgi may play critical roles in viral assembly and egress, protein trafficking, apoptosis [37], [38], and the cellular stress response [38]. Accordingly, the recently defined role of pUL138 in modulating surface levels of TNFR, suggests that pUL138 may mediate protein trafficking [20], [21] and, therefore, the cellular response to signaling molecules.

The conclusions drawn from our *in vitro* studies are bolstered by our *in vivo* studies in humanized mice. The increased viral loads of TB40E-*UL133-UL138*_NULL_ virus in the spleens of NOD-*scid* IL2Rγ_c_^null^-humanized mice following mobilization suggests increased replication, reactivation or dissemination of this virus relative to the WT virus (Figure 9B). This *in vivo* finding further supports an important role for the *UL133-UL138* locus in suppressing replication or reactivation for latency. Mobilization did not significantly increase WT or *UL133-UL138*_NULL_ viral genome copy number in the bone marrow (Figure 9A), possibly reflecting the fact that mobilized cells quickly exit the bone marrow. The fact that higher genome levels were not measured in the bone marrow of *UL133-UL138*_NULL_-infected mice relative to WT-infected mice prior to mobilization, suggests that these viruses may not behave differently in this system in the absence of a reactivation stimulus. The nature of the humanized mice studies is such that the results cannot completely recapitulate our *in vitro* studies, yet they are highly consistent with our *in vitro* studies, both studies suggesting an important role for the *UL133-UL138* locus in modulating the outcomes of infection. Future studies into the *UL133-UL138* locus promise to reveal intriguing virus-host interactions unique to higher-order primates mediating viral persistence.

## Materials and Methods

### Ethics statement

Human cord blood was obtained from donors at the University Medical Center at the University of Arizona using a protocol approved by the Institutional Review Board. These specimens are completely deidentified and provided to our research group as anonymous samples. The studies requiring animals were carried out in strict accordance with the recommendations of the American Association for Accreditation of Laboratory Animal Care (AAALAC). The protocol was approved by the Institutional Animal Care and Use Committee (number IS00001049) at Oregon Health and Science University.

### Cells

Human embryonic lung fibroblasts (MRC5) (purchased from ATCC; Manassas, VA) were cultured at 37 °C in Dulbecco's modified Eagle's medium (DMEM) supplemented with 10% fetal bovine serum (FBS), 10 mM HEPES, 1 mM sodium pyruvate, 2 mM L-Glutamine, 0.1 mM Non-essential amino acids, 100 U/ml penicillin and 100 µg/ml streptomycin. Human cord blood was obtained from donors at the University Medical Center at the University of Arizona using a protocol approved by the Institutional Review Board. These specimens are completely deidentified and provided to our research group as anonymous samples. Mononuclear cells and CD34^+^ HPCs were isolated and cultured as previously described [19], [22]. CD34^+^ cells were maintained in long-term culture as described previously [19] but using MyeloCult H5100 (Stem Cell Technologies). The M2-10B4 murine stromal cell line expressing human interleukin-3 (IL-3) and granulocyte-colony stimulating factor (G-CSF) and the S1/S1 murine stromal cell line expressing human IL-3 and stem cell factor (SCF) (kind gift from Stem Cell Technologies on behalf of D. Hogge, Terry Fox Laboratory, University of British Columbia, Vancouver, BC) and cultured as recommended [39]. Primary human microvascular lung endothelial cells (HMVEC), human umbilical vein endothelial cells (HUVEC), human umbilical vein endothelial cells (HUVEC), and human aortic endothelial cells (HUAEC) were purchased from Lonza (Walkersville, MD). HMVEC, HUAEC, HUVEC cells were cultured in EGM-2 MV (Microvasular Endothelial Cell Growth Medium-2; Lonza), EGM-MV (Endothelial Growth Medium- MV; Lonza) and EGM (Endothelial Growth Medium; Lonza). Human aortic endothelial cells (HAEC) were a generous gift from Andrew Yurochko and were cultured in EGM-2 (Endothelial Growth Medium-2; Lonza). Human renal cortical epithelial cells (HRCE) were purchased from Lonza (Walkersville, MD) and were cultured in REGM (Renal Epithelial Cell Growth Medium; Lonza). All cells were maintained at 37°C with 5% CO_2_.

### Viruses

Recombinant bacterial artificial chromosomes (BACs) containing the HCMV genome were constructed in *Escherichiae coli* (*E. coli*) by linear recombination in a two-step positive-negative selection method that leaves no trace of the engineering process [19], [40], [41]. The green fluorescent protein (GFP) was engineered between US34 and TRS1 in BAC clones of FIX [42], [43] or TB40E [44] virus strains as a marker of infection. In the first step, the SW102 *E. coli* strain containing the FIX or TB40E BAC were used to insert a *galk* cassette between US34 and TRS1 genes. In the second step, the *galk* cassette was replaced by an SV40-eGFP-BGH Poly-A cassette PCR amplified from the pCMS-eGFP vector (Clonetech) to generate the FIX or TB40E BACs used as the parental wild type strains in all experiments herein. These variants replicate with kinetics and to titers identical to the parental strains (data not shown). Further recombinant viruses were generated by repeating the insertion and substitution of *galk* using a PCR product flanked by homologous viral sequences as described previously [19], [22]. Oligonucleotide primers used for BAC recombineering are described in Table 1. Recombinant viruses were screened by BAC digestion, PCR, and sequencing. Virus stocks were propagated, stored and titered as described previously [19].

**Table 1:** Primers used to generate recombinant viruses.

Primer name	Sequence (5′ to 3′)
FIX or TB40E-GFP-GalK-Fwd	CGTGTCCTGGTTTTTCATTTTTTGGATGTATTTGTCGCA
	TAAAAGGCGGTCCTGTTGACAATTAATCATCGGC
FIX or TB40E-GFP-GalK-Rev	TGTTAGGATAACAAAACTGCGTATCTGGATATATTTCA
	TCCCCACATCCCTCAGCACTGTCCTGCTCC
FIX or TB40E-GFP--Fwd	CGTGTCCTGGTTTTTCATTTTTTGGATGTATTTGTCGCA
	TAAAAGGCGGTGCAGCACCATGGCCTGAAAT
FIX or TB40E-GFP-Rev	TGTTAGGATAACAAAACTGCGTATCTGGATATATTTCA
	TCCCCACATCCCACCATACGCGGATCTGCC
TB40E-UL133-UL138_NULL_-Fwd	ACTCGGCAGCCACTGTAGGGATAAATAGTGCGATGGC
	GTTTGTGAGAGAACGCCTGTTGACAATTAATCATC
TB40E-UL133-UL138_NULL_-Rev	TTCATTCTGGGGTTTCCCAATGACGTAAAAATTTCCAC
	TACACAATAAAATCAGCACTGTCCTGCTCCTT
T40E-UL133_Stop_-GalK-Fwd	CCACTGTAGGGATAAATAGTGCGATGGCGTTTGTGAG
	AGAACGCAGTAGCGCCTGTTGACAATTAATCATCGGCA
T40E-UL133_Stop_-GalK-Rev	GGAAGGAGATGTGGGCCAAGTCGGAAAATTCCTTATC
	ACACCGGGGGCGGGTCAGCACTGTCCTGCTCCTT
T40E-UL133_Stop_-Fwd	CCACTGTAGGGATAAATAGTGCGATGGCGTTTGTGAGA
	GAACGCAGTAGCGTAAGGTTGAGACGTGCACGATCCTTCG
T40E-UL133_Stop_-Rev	GGAAGGAGATGTGGGCCAAGTCGGAAAATTCCTTATCA
	CACCGGGGGCGGGTTACGTTCCGGTCTGATGCTGCTGCTG
T40E-UL138_Stop_-GalK-Fwd	CCATGGACGATCTGCCGCTGAACGTCGGGTTACCCATCA
	TCGGCGTGCCTGTTGACAATTAATCATC
T40E-UL138_Stop_-GalK-Rev	TCGTGCCAATGGTAAGCTAGATAGCAGAGAATGGCCAC
	GATCAGCACGAGTCAGCACTGTCCTGCTCCTT
T40E-UL138_Stop_-Fwd	CCATGGACGATCTGCCGCTGAACGTCGGGTTACCCAT
	CATCGGCGTGTAACTCGTGCTGATCGTGGCCATTCTC
	TGCTATCTAGCTTACCATTGGCACGA
T40E-UL138_Stop_-Rev	TCGTGCCAATGGTAAGCTAGATAGCAGAGAATGGCCA
	CGATCAGCACGAGTTACACGCCGATGATGGGTAACCC
	GACGTTCAGCGGCAGATCGTCCATGG

### Plasmids

Oligonucleotide primers used for making expression plasmids are described in Table 2. The *UL133*, *UL135* and *UL136* ORFs were PCR amplified using ORF specific primers flanked by a NheI site on the forward primer and a BamHI site on the reverse primer. The reverse primer contained the myc epitope tag sequence to generate 3′ tagged versions of each gene. The PCR products were cloned into the NheI and BamHI sites of the pCIG2 vector [22]. These constructs termed pCIG-UL133_myc_, pCIG-UL135_myc_, and pCIG-UL136_myc_, express proteins with a C-terminal myc epitope tag with a 5 amino acid linker between the protein coding sequence and the myc tag. To obtain HA (YPYDVPDYA), 3X-FLAG (DYKDDDDK), or Glu-Glu (E-E) (EYMPME) (at the C-terminus) versions of these proteins, the PCR products containing a specific epitope tag were cloned into NheI and EcoRV sites of pCIG2 vector as above. The resulting plasmids were named pCIG2-UL133_FLAG_-IRES-BLEO, pCIG2-UL135_HA_-IRES-HYGRO, pCIG2-UL136_myc_-IRES-NEO, and pCIG2-UL138_EE_-IRES-PURO. In these constructs, the eGFP downstream of IRES was replaced by drug resistance markers such as Hygromycin (HYGRO), Neomycin (NEO), Bleomycin (BLEO) or Puromycin (PURO).

**Table 2:** Primers used for cloning into pCIG2 vector.

Primer name^a^	Sequence (5′ to 3′)^b^
UL133-Fwd	*ccggaattcgctagcaccATGGGTTGCGACGTGCACGATCCTTCG*
UL135-Fwd	cgcggatccgctagcaccATGTCCGTACACCGGCCCTTCCCGACA
UL136-Fwd	ccggaattcgctagcaccATGTCAGTCAAGGGCGTGGAGATGCCA
UL138-Fwd	ggggGCTAGCACCATGGACGATCTGCCGCTGAACGTCG
C-Myc-Rev	cgcggatccTCACAGATCCTCTTCTGAGATGAGTTTTTGTTC
UL133_3xFLAG_-Rev	ggggGATATCTTACTTGTCGTCGTCGTCCTTGTAGTCGAATTCCTTGTC
	GTCGTCGTCCTTGTAGTCTGCCCCTTTATCATCATCATCTTTATAATC
	ACCGCCACCGCCCGTTCCGGTCTGATGC
UL135_HA_-Rev	gggggatatcTCACGCGTAATCTGGAACATCGTATGGGTAACCGCCAC
	CGCCGGTCATCTGCATTGACTCGGCGTCC
UL136_myc_-Rev	gggggatatcTTACAGATCCTCTTCTGAGATGAGTTTTTGTTCACCGCC
	ACCGCCCGTAGCGGGAGATACGGCGTTCTCC
UL138_EE_-Rev	gggggatatcTCACTCCATGGGCATGTACTCACCGCCACCGCCCGTGTA
	TTCTTGATGATAATGTACC
Bleo-Fwd	ggggaccggtaccATGGCCAAGTTGACCAGTGCCGTTCCG
Bleo-Rev	gggggcggccgcTCAGTCCTGCTCCTCGGCCACG
Neo-Fwd	ggggaccggtaccATGGGATCGGCCATTGAACAAGATGGATTGC
Neo-Rev	gggggcggccgcTCAGAAGAACTCGTCAAGAAGGCGATAGAAGG
Puro-Fwd	ggggaccggtaccATGACCGAGTACAAGCCCACGGTGCG
Puro-Rev	gggggcggccgcTCAGGCACCGGGCTTGCGGGTC
Hygro-Fwd	ggggaccggtaccATGGATAGATCCGGAAAGCCTGAACTCACC
Hygro-Rev	gggggcggccgcCTATTCCTTTGCCCTCGGACGAGTGC

a Fwd: Forward; Rev: Reverse

b Enzyme sites are in lower case

### Lentivirus production

The plasmids were co-transfected with pLP1, pLP2 and pVSVG plasmids (Invitrogen, CA) at 2∶1∶1∶1 ratio into 293FT cells using Lipofectamine 2000 (Invitrogen, CA). Culture supernatants were harvested 48 hpi and concentrated at 17,000 rpm using a SW28 rotor for 2 h at 4^o^C. Pellets were resuspended in IMDM containing 10% BIT9500 (Stem Cell technologies). Lentiviruses were titered on fibroblasts using the TCID_50_ method.

### Immunoblotting

Immunoblotting was performed as described previously [19]. Briefly, 10–15 µg of protein lysates were separated on 4–12% NuPAGE Bis-Tris, (Invitrogen, CA) or 11% Bis-Tris gels by electrophoresis and transferred to 0.45 µm polyvinylidene difluoride (Immobilon-FL, Millipore, MA) membranes. The proteins were immunoblotted using mouse α-myc (Cell Signalling) or rabbit polyclonal antibodies directed against each protein (Open Biosystems) and detected using fluorescently conjugated secondary antibodies and the Odyssey infrared imaging system (Li-Cor, NE). All antibodies used are listed in Table 3.

**Table 3:** Primary antibodies used for immunofluorescence and immunoblotting.

Antigen	Antibody	Type^a^	Source	Dilution	
				Immunoflurescenceb,d,g	Immunoblottingc,d
UL133	Custom	R	Open Biosystems	ND	2 µg/ml
UL135	Custom	R	Open Biosystems	ND	2 µg/ml
UL138	Custom	R	Open Biosystems	ND	2 µg/ml
IE1/2	3H4	M	*Gift* e	ND	1∶100
MHC-I, HLA-B/C	HC10	M	Gift^f^	ND	1∶50
α-Tubulin	DM 1A	M	Sigma	ND	1∶12,000
β-Actin	ACTN05(C4)	M	abcam	ND	1∶1000
Golgi GM130	Clone 35	M	BD Transduction	1∶100	ND
			Laboratories		
Myc epitope	71D10	R	Cell Signalling	1∶200	1∶1000
Myc epitope^g^	9B11	M	Cell Signalling	1∶1000	1∶1000
HA epitope^g^	HA7	M	Sigma	1∶100	ND
FLAG epitope^g^	M2	M	Sigma	1∶2000	1∶1000
EE epitope^g^		R	Bethyl	1∶1000	ND
			Laboratories, Inc		

a R-Rabbit; M-Mouse monoclonal

b Dilution in PBS supplemented with BSA and Tween 20

c Dilution in TBS/5% milk supplemented with BSA and Tween 20

d ND-Not done

e Generous gift from Tom Shenk, Princeton University.

f Generous gift from Lonnie Lybarger, University of Arizona.

g Conjugated with Qdots 525nm (HA); 565nm (myc); 585nm (FLAG); 625nm (EE)

### Indirect immunofluorescence

Immunofluorescence to localize viral and cellular proteins in infected cells was performed as described previously [19]. Briefly, fibroblasts (5×10^4^ cells/well in 24-well plates) were mock infected or infected with recombinant viruses encoding myc epitope-tagged pUL133, pUL135, pUL136 and pUL138 at an MOI of 2 for 24 and 48 h. Cells were fixed in 2% paraformaldehyde in PBS and stained with a rabbit antibody specific to myc epitope tag and visualized using a Zeiss 510 Meta confocal microscope (Carl Zeiss Microimaging, Inc. NY). The nucleus was stained with 1 µg/ml DAPI (4′, 6′-diamidino-2- phenylindole) and GM130 was used as a Golgi marker.

### Direct immunofluorescence

Fibroblasts were seeded on to 12 mm glass cover slips in 24 well plates one day prior to infection. The next day cells were mock infected or infected with TB40E-*UL133-UL138*_NULL_ at an MOI of 2. At 6 hpi, cells were transduced with lentiviruses containing pCIG2-UL133_FLAG_-IRES-BLEO, pCIG2-UL135_HA_-IRES-HYGRO, pCIG2-UL136_myc_-IRES-NEO, pCIG2-UL138_EE_-IRES-PURO alone or in combination in the presence of 8 µg/mL of Polybrene (Sigma-Aldrich, St. Louis, MO). Cells were processed for direct immunofluorescence 48 hours later using the same method used for indirect immunofluorescence except for the primary antibody incubations, nuclear staining, and mounting. Briefly, primary antibodies to the various epitope tags were conjugated to amine derivatized Quantum Dots (Molecular Probes, Invitrogen, Carlsbad, CA) of 525 nm (Anti-HA), 565 nm (Anti-myc), 585 nm (Anti-FLAG), and 625 nm (Anti-EE) emission wavelengths according to manufacturer's instructions (Table 3). Primary antibodies were incubated in PBS-T + 1% BSA overnight at 4°C. Post staining, cells were washed 3 times in PBS-T and nuclei stained with Qnuclear Deep Red Stain (Molecular Probes, Invitrogen, Carlsbad, CA) according to manufacturer's instructions. Coverslips were washed 3 times in PBS and mounted using Qmount Qdot mounting media (Invitrogen) according to manufacturer's instructions. Cells were imaged using a Ziess 510 Meta Confocal Microscope as a lambda stack and unmixed using the Zeiss 510 Meta software version 4.2 and images were processed using ImageJ software, NIH (http://rsbweb.nih.gov/ij/download.html).

### Membrane fractionation and association

Microsome preparation was done as described previously [19]. Briefly, fibroblasts were either mock infected or infected with FIX-UL136_myc_ virus at an MOI of 1 for 48 h. Cells were treated with 50 µg/ml cycloheximide for 4 h prior to harvesting at 48 h post infection. Cells were resuspended in buffer A (250 mM Sucrose, 50 mM triethanolamine, 1 mM EDTA, 6 mM magnesium acetate, 50 mM potassium acetate and 1 mM dithiothreitol) and gently sonicated 3 times for 10 sec on ice at 30 sec intervals. Membranes were fractionated by differential centrifugation to obtain membrane pellets at 3000×*g*, 12,000×*g*, 25,000×*g* and 100,000×*g*. The supernatant obtained post 100,000×*g* was precipitated using trichloroacetic acid (TCA). All pellets were resuspended in identical volumes of buffer A and analyzed by immunoblotting using protein specific antibodies (Table 3).

For salt extraction, the 25,000×*g* membrane fractions were treated with 100 mM Na_2_CO_3_ for 1h on ice and centrifuged at 100,000×g in a TLA 100.3 rotor for 45 min at 4°C to separate the pellet and supernatant, subsequently precipitated using TCA. Equal amounts of input, pellet and supernatant were analyzed by immunoblotting as above.

To determine the topology of proteins in the membrane, the 25,000×*g* membrane fractions were digested with 0.5 µg/ml proteinase K in the absence or presence of 1% Triton X-100 for 1 hr at 37^o^C. Reactions were stopped by adding 1 mM PMSF and SDS sample buffer. Input and protease treated samples were analyzed by immunoblotting.

### Quantitative reverse transcriptase PCR

Targets were detected by qRT-PCR (Quantitative reverse-transcription PCR) as described previously [22] **.** RNA was isolated from TB40E-WT (Sample) or TB40E-*UL133-UL138*_NULL_ (Control) infected cells and DNase treated using the NucleoSpin RNA II kit (Machery-Nagel) and cDNA was generated using the transcriptor first-strand cDNA synthesis kit (Roche) according to the manufacturer's instructions. qRT-PCR was performed with the LightCycler 480 Probes Master (Roche) according to the manufacturer's instructions along with the Universal Probe Library (Roche) probes and primers specific for *UL133*, *UL135*, *UL136* and *UL138* [22]. IE1&2 genes were used as controls for infection. The human β-actin gene was used as a reference and the target levels were quantitated by a ΔΔCT method using the following equation [45].
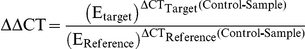



Where, CT is the cycle threshold and E is the efficiency as determined using the Light Cycler 480 software. In our analysis, we considered a 2-fold change to be within the confidence interval for equally abundant target.

### Infectious centers assay

CD34^+^ HPCs isolated from human cord blood were infected at an MOI of 2 for 20 h in IMDM supplemented with 10% BIT9500 serum substitute (Stem Cell Technologies, Canada), 2 mM L-Glutamine, 20 ng/ml low density lipoproteins and 50 µM 2-mercaptoethanol. Following infection, pure populations of infected CD34^+^ HPCs (GFP-positive) were isolated by fluorescence-activated cell sorting (FACSAria, BD Biosciences Immunocytometry Systems, San Jose, CA) using a phycoerythrin-conjugated antibody specific to CD34 (BD Biosciences) and cultured in transwells above an irradiated (4000 rads, ^137^Cs gammacell-40 irradiator type B, Atomic Energy of Canada LTD, Ottawa, Canada) M2-10B4 and S1/S1 stromal cell monolayer [39] for 10–12 days. The frequency of infectious centers production during the culture period was measured using a limiting dilution assay as described previously [18]. Briefly, infected HPCs were serially diluted 2-fold in LTBMC medium. An aliquot (0.05mL) of each dilution was added to 12 wells (first dilution corresponds to 40,000 cells per well) of a 96-well tissue culture plates containing MRC5 cells. MRC5 cells were monitored for GFP expression for a period of 14 days. The frequency of infectious centers formed was calculated based on the number of GFP^+^ cells at each dilution using software, Extreme limiting dilution analysis (ELDA, http://bioinf.wehi.edu.au/software/elda) [46].

### Engraftment and infection of humanized mice

*NOD-scid IL2Rγc^null^* mice were maintained in a specific pathogen free facility at Oregon Health and Science University in accordance with Institutional Animal Care and Use Committee approved procedures. Mice were sublethally irradiated with 250 cGy by ^137^Cs g-irradiation and then engrafted with approximately 150,000 human CD34^+^ stem cells (Catalog #CB008F-S, StemCell Technologies; Vancouver British Columbia, Canada) via retro-orbital injection. At 4 weeks post inoculation of stem cells, the level of engraftment (determined as the percentage of human CD45^+^ present in the blood of total lymphocytes) was assessed by flow cytometry as previously described [27]. At 5 weeks post engraftment, the mice were infected by intraperitoneal injection of 1×10^7^ normal human dermal fibroblasts previously infected with HCMV TB40E-WT or TB40E-*UL133-UL138*_NULL_. A third group of engrafted mice were mock infected with uninfected human fibroblasts. At 4 weeks post infection, a group of mice were treated for 7 days with 100 µl of G-CSF (300 µg/ml; Amgen) via a subcutaneous micro-osmotic pump (1007D; Alzet) and AMD3100 (5mg/kg) to mobilize their hematopoietic stem cells. As a direct comparator for the effects of virus reactivation-dissemination following mobilization an additional non-mobilized infected control group was included for each virus (n = 4/group). At 2 weeks post mobilization, mice were euthanized and bone marrow and spleen were harvested and snap frozen for subsequent analysis.

### Quantitative PCR for viral genomes

Total DNA was extracted from 0.8 g of mouse spleen and bone marrow via the DNAzol method (Life Technologies) and analyzed by quantitative PCR (TaqMan) for the presence of HCMV genomic DNA. For Q-PCR analysis, 1 µg of total DNA was analyzed using primers and a probe recognizing HCMV US28 sequences (probe = TGA TCC CGC TCA GTG T; forward primer = GAA CTC ATG CTC GGT GCT TTC; and reverse primer = CTT TGT GGC GCG ACT GAG A). The probe contains labeled with a 5′-end FAM reporter molecule and a 3′-end quencher molecule (Applied Biosystems, Foster City, CA). PCR reactions were prepared using TaqMan Universal PCR Master Mix (Applied Biosystems) according to the manufacturer's instructions. To initiate the reaction the AmpliTaq Gold was activated at 95°C for 10 minutes and then 40 cycles (15 s at 95°C and 1 min at 60°C) were performed using a StepOnePlus TaqMan PCR machine. Results were analyzed using ABI StepOne software. The sensitivity of detection for this assay was approximately 50 HCMV DNA genomic copies as determine by using a plasmid containing the US28 amplicon to develop a standard curve. Data were analyzed using the statistical program GraphPad Prism 5.

## Supporting Information

Figure S1 **Virus replication of** **FIX recombinant viruses at high MOI.** Cells and media were collected 6 dpi following MRC5 infection with FIX-UL133_myc_, FIX-UL135_myc_, FIX-UL136_myc_ and FIX-UL138_myc_ recombinant viruses. Virus titers were measured by TCID_50_. Bars represent the average of triplicate experiments.
(EPS)

Figure S2 **Transiently expressed pUL133, pUL135, and pUL136 associate with microsomal membranes.** (A) To determine the membrane association of each viral protein in the absence of virus infection, MRC5 cells were transduced with lentiviruses expressing pUL133_myc_ pUL135_myc_ pUL136_myc_ and pUL138_myc_ at an MOI of 1. Crude membrane fractions were prepared at 48 hours post transduction by differential centrifugation as described in Figure 3A. To determine if the viral proteins were integral membrane proteins, 25K microsomal membrane fractions were treated with 100 mM Na_2_CO_3_ for 1 h on ice and pelleted at 100,000×g for 1 h at 4^o^C. Equal amounts of input, pellet and supernatant were analyzed for specific proteins by immunoblotting using a rabbit antibody specific to myc epitope tag. (B) To determine the topology of proteins in the Golgi membranes, 25K microsomal membrane fractions were treated with 0.5 µg/ml proteinase K for 45 min at 37°C in the presence or absence of 1% Triton X-100. Viral proteins were detected by immunoblotting as described above**.** MHC I was used as a control in panels A-B. The bands were quantified using Odyssey LiCor software and the percent protein digestion in the absence or the presence of Triton X-100 is indicated.
(EPS)

Figure S3 ***UL133-UL138*** **_NULL_ virus replication with high MOI infection.** To examine the replication kinetics of the *UL133-UL138*_NULL_ viruses relative to the wild type viruses at high MOI, MRC5 cells were infected with (A) TB40E-*UL133-UL138*_NULL_ or (B) FIX-*UL133-UL138*_NULL_ at 2 MOI and virus titers measured by TCID_50_ at 6 dpi. Bars represent the averages of triplicate experiments.
(EPS)

Figure S4 **Localization of pUL133, pUL135, pUL136 and pUL138 in the Golgi apparatus.** MRC5 cells were mock-infected or infected with TB40E-*UL133-UL138*_NULL_ and then transduced with lenti viruses encoding pUL133_FLAG_, pUL135_HA_, pUL136_myc_, or pUL138_EE_. Proteins were co-localized by direct immunofluorescence 48 hpi using monoclonal antibodies specific to each epitope tag that had been directly conjugated to Quantum dots of 525nm (HA); 565nm (Myc); 585nm (FLAG); 625nm (EE). Cell nuclei are indicated by Qnuclear staining. Localization was visualized using a Ziess 510 Meta Confocal microscope.
(TIF)
