# Supplementary figures and images for: A Novel Human Cytomegalovirus Locus Modulates Cell Type-Specific Outcomes of Infection
Source: PLoS Pathog. 2011 Dec 29;7(12):e1002444. doi: 10.1371/journal.ppat.1002444 (PMC3248471; doi:10.1371/journal.ppat.1002444)

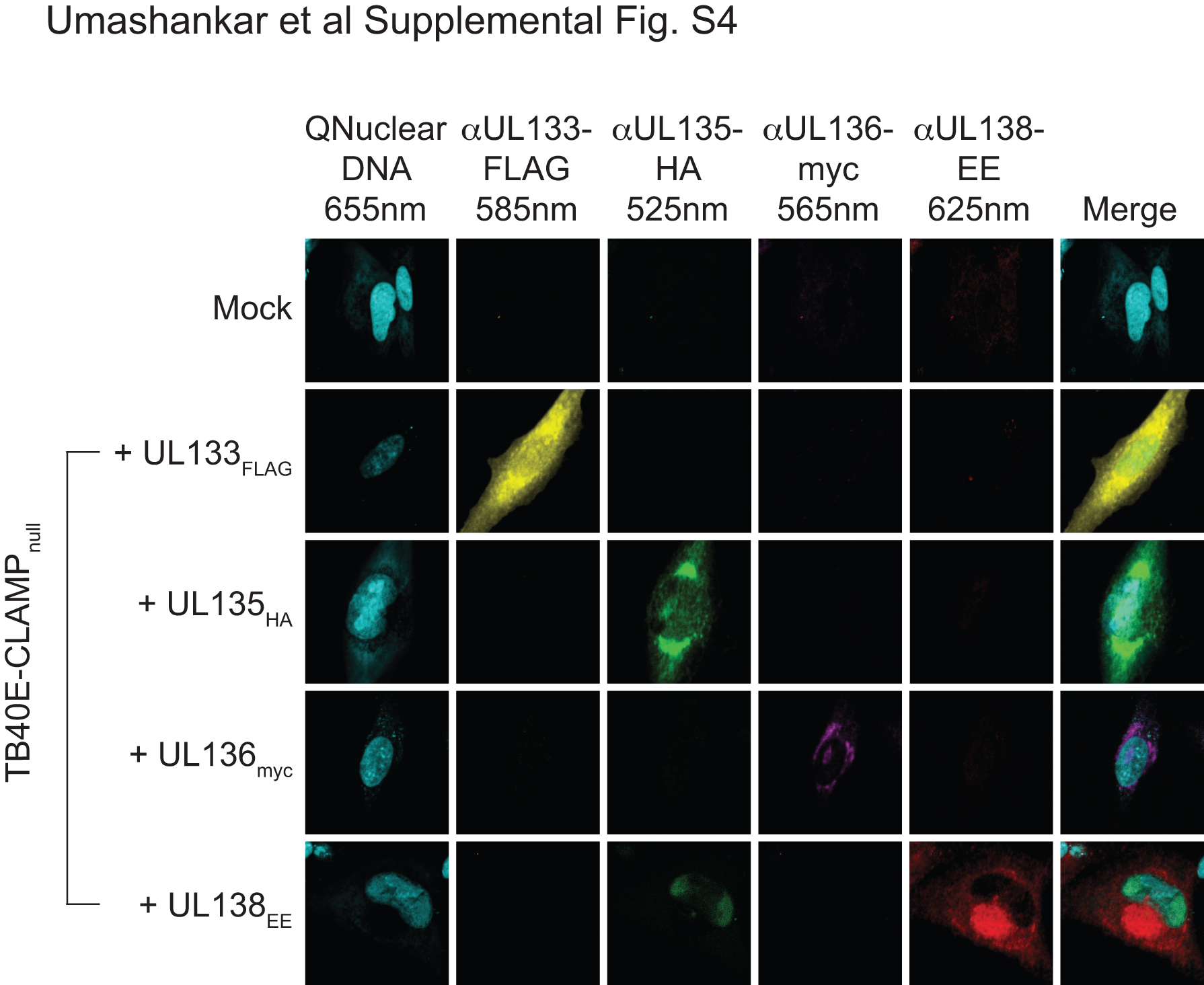

Supplement: Figure S4 — Localization of pUL133, pUL135, pUL136 and pUL138 in the Golgi apparatus. MRC5 cells were mock-infected or infected with TB40E-UL133-UL138 NULL and then transduced with lenti viruses encoding pUL133FLAG, pUL135HA, pUL136myc, or pUL138EE. Proteins were co-localized by direct immunofluorescence 48 hpi using monoclonal antibodies specific to each epitope tag that had been directly conjugated to Quantum dots of 525nm (HA); 565nm (Myc); 585nm (FLAG); 625nm (EE). Cell nuclei are indicated by Qnuclear staining. Localization was visualized using a Ziess 510 Meta Confocal microscope. (TIF) [file ppat.1002444.s004.tif]
